# Poly-epigenetic scores for cardiometabolic risk factors interact with demographic factors and health behaviors in older US Adults

**DOI:** 10.1080/15592294.2025.2469205

**Published:** 2025-02-20

**Authors:** Lisha Lin, Wei Zhao, Zheng Li, Scott M. Ratliff, Yi Zhe Wang, Colter Mitchell, Jessica D. Faul, Sharon L. R. Kardia, Kira S. Birditt, Jennifer A. Smith

**Affiliations:** a Department of Epidemiology, School of Public Health, University of Michigan, Ann Arbor, MI, USA; b Survey Research Center, Institute for Social Research, University of Michigan, Ann Arbor, MI, USA; c Department of Biostatistics, School of Public Health, University of Michigan, Ann Arbor, MI, USA

**Keywords:** Poly-epigenetic score, DNA methylation, cardiometabolic risk factors, blood pressure, body mass index, inflammation, blood lipids, glucose

## Abstract

Poly-epigenetic scores (PEGS) are surrogate measures that help capture individual-level risk. Understanding how the associations between PEGS and cardiometabolic risk factors vary by demographics and health behaviors is crucial for lowering the burden of cardiometabolic diseases. We used results from established epigenome-wide association studies to construct trait-specific PEGS from whole blood DNA methylation for systolic and diastolic blood pressure (SBP, DBP), body mass index (BMI), C-reactive protein (CRP), high- and low-density lipoprotein cholesterol (HDL-C, LDL-C), triglycerides (TG), and fasting glucose. Overall and race-stratified associations between PEGS and corresponding traits were examined in adults >50 years from the Health and Retirement Study (*n* = 3,996, mean age = 79.5 years). We investigated how demographics (age, sex, educational attainment) and health behaviors (smoking, alcohol consumption, physical activity) modified these associations. All PEGS were positively associated with their corresponding cardiometabolic traits (*p* < 0.05), and most associations persisted across all racial/ethnic groups. Associations for BMI, HDL-C, and TG were stronger in younger participants, and BMI and HDL-C associations were stronger in females. The CRP association was stronger among those with a high school degree. Finally, the HDL-C association was stronger among current smokers. These findings support PEGS as robust surrogate measures and suggest the associations may differ among subgroups.

## Introduction

Cardiovascular diseases (CVD) are global leading causes of mortality [1], imposing a substantial health burden among US adults [2,3]. The 2017 to 2020 National Health and Nutrition Examination Survey (NHANES) underscored a markedly elevated prevalence of CVD among those aged 60 years and above compared to younger participants [4]. Major cardiometabolic risk factors that contribute to CVD development include hypertension, diabetes, dyslipidemia, overweight and obesity, and inflammation [5–9]. Despite advancements in healthcare and treatment leading to longer life spans in recent decades, the prevalence of these cardiometabolic risk factors continues to rise rapidly [9–14], and their burden is disproportionate across races/ethnicities [15–17]. Therefore, it is essential to continue to refine strategies for prevention and early treatment of these risk factors to lower cardiometabolic burden and reduce health disparities.

Recently, there is a growing interest in utilizing epigenome-based surrogate measures to further unravel the intricate relationship among epigenetics, aging, and cardiometabolic risk factors. Most epigenetic studies focus on DNA methylation, which occurs at cytosine-guanine (CpG) sites across the genome in which a methyl group is added to a cytosine base [18]. DNA methylation acts as a regulator of transcription without altering the DNA sequence [19], and it may be modifiable [20]. The availability of epigenome-wide association studies (EWAS) has enabled the development of poly-epigenetic scores (PEGS), also known as methylation risk scores (MRS), which aggregate methylation levels across a subset of CpG sites that are highly associated with specific traits of interest [21] to construct a summary measure of epigenetic ‘risk’ for the trait. Other epigenetic-based markers, such as epigenetic clocks that capture biological aging [22], may also be associated with cardiometabolic risk factors.

To our knowledge, few studies have examined the associations between PEGS and cardiometabolic risk factors in a multi-racial/ethnic sample, although some have been examined in single racial/ethnic groups. For example, a PEGS for BMI constructed in a European ancestry sample was associated with higher BMI and greater body weight in a second European ancestry sample [23]. Moreover, methylation at CpGs has been associated with cardiometabolic risk factors, including diabetes [24] and obesity [25]. For example, a study involving 280 Swedish participants used PEGS as indicators to identify differential epigenetic patterns among four subgroups of type-2 diabetes [26]. Therefore, it is important to better understand how the interindividual ‘risk’ of having unfavorable cardiometabolic risk factors may be reflected in epigenetic surrogates, such as PEGS, and whether these associations differ by demographic or health behavior groups. In this study, we constructed PEGS for eight cardiometabolic risk factors in a diverse, nationally representative sample of older adults in the US. We then tested whether the association between the PEGS and its associated cardiometabolic trait varied by demographic factors (age, sex, educational attainment) and behavioral factors (smoking, alcohol consumption, physical activity). This study aims to provide insights into the potential of advancing precision health by identifying subgroups wherein these scores might perform better based on individual demographic characteristics or health behaviors.

## Methods

### Study sample

The Health and Retirement Study (HRS) is a US nationally representative longitudinal cohort of over 20,000 adults over age 50 years and their spouses/partners. The initial interview was conducted in 1992, with a follow-up every 2 years. An ancillary study, the 2016 Venous Blood Study (VBS) (*N* = 9,934), collected biomarkers for cardiometabolic risk factors such as CRP, blood lipids, and fasting glucose [27]. VBS participants were encouraged but not required to fast prior to the blood draw. HRS then sampled 4,104 respondents from the 2016 VBS study and performed DNA assay measurements. Participants aged 55 and above with available DNA methylation (*n* = 3,963) were assigned sample weights, which adjusted for the differential probabilities of participation for age-eligible respondents [28]. The weighted sample represents adults 55 years and above in the US All participants provided signed informed consent before participation. Following the exclusion of participants with missing self-reported race/ethnicity (*n* = 3), alcohol use status (*n* = 18), smoking status (*n* = 1), and educational attainment (*n* = 1), a total of 3,996 participants were included in the main analysis and 3,855 participants were included in the analysis using sample weights.

### Cardiometabolic risk factors

We assessed eight cardiometabolic risk factors: systolic and diastolic blood pressure (SBP/DBP), body mass index (BMI), CRP, low-density lipoprotein cholesterol (LDL-C), high-density lipoprotein cholesterol (HDL-C), triglycerides (TG), and fasting glucose. Measurement details for the cardiometabolic risk factors are described below. For each risk factor, outliers exceeding 5 standard deviations (SD) from the mean were windsorized for statistical purposes.

### Systolic and diastolic blood pressure (SBP/DBP) (mmHg)

Blood pressure was assessed using an Omron BP 760 N Monitor (Omron Healthcare, Bannockburn, IL, USA) during face-to-face interviews in 2016 (*n* = 1,829) or 2018 (*n* = 1,614). Each measurement was taken 45 seconds apart on the participant’s left arm. To ensure data accuracy, measurements of SBP that exceeded 250 mmHg and DBP below 40 mmHg were censored. Subsequently, the mean of the remaining blood pressure measurements was calculated for each participant.

### Body mass index (BMI) (kg/m^2^)

Height and weight were obtained from the concurrent BP measurement wave (2016 or 2018). BMI was calculated using the participant’s weight in kilograms divided by the square of the height in meters.

### C-reactive protein (CRP) (mg/L)

CRP levels were measured in serum using a latex-particle enhanced immunoturbidimetric assay kit (Roche Diagnostics, Indianapolis, IN 46,250) and read on the Roche COBAS 6000 Chemistry analyzer (Roche Diagnostics). Prior to analysis, CRP values were natural log-transformed.

### Blood lipid measures (mg/dL)

Three blood lipid measures (LDL-C, HDL-C, and TG) were included in this study. While participants were encouraged to fast before providing blood samples, it was not mandatory. All blood lipids were measured in serum using a Roche Cobas 6000 Chemistry Analyzer (Roche Diagnostics Corporation, Indianapolis, IN) [27]. Total cholesterol (TC) was measured using the cholesterol oxidase method. HDL-C was assessed using the Roche HDL-Cholesterol 3rd generation direct method. TG was measured using an enzymatic Triglyceride Reagent. LDL-C was calculated for participants with TG levels less than 400 mg/dL using Friedewald’s formula: LDL (mg/dL) = TC – HDL-C – (TG/5.0) [29]. Both HDL-C and TG were natural log-transformed prior to analysis.

### Fasting glucose (mg/dL)

Glucose was measured on a Roche Cobas 6000 Chemistry Analyzer (Roche Diagnostics Corporation) using the Roche hexokinase method (Roche Diagnostics, Indianapolis, IN). Because blood glucose levels are highly dependent on fasting status, we limited glucose measures to those who self-reported fasting prior to the blood draw (*n* = 2,628).

### DNA methylation measurement

DNA methylation was measured in whole blood samples using the Infinium MethylationEPIC BeadChip for 4,104 VBS participants. Samples were randomized across plates by key demographic variables including age, cohort, sex, education, and race/ethnicity with 40 pairs of blinded duplicates [28]. Normal-exponential out-of-band background correction and dye-bias normalization were performed for data preprocessing using the *preprocessNoob ()* function. Probes with a detection *p*-value >0.01 were removed. Samples with >5% missing probes or mismatched sex, as well as control samples, were excluded [28]. Both data preprocessing and quality control were conducted in R software with the *minfi* package. The final analytical sample for DNA methylation consists of 4,018 participants.

### Poly-epigenetic scores (PEGS)

PEGS for a given individual are generally constructed as the weighted sum of their methylation beta values at a list of CpGs identified from an epigenome-wide association study (EWAS) [21]. In this study, PEGS were calculated for eight cardiometabolic risk factors (SBP, DBP, BMI, CRP, LDL-C, HDL-C, TG, fasting glucose). For each risk factor, an EWAS was selected by prioritizing those with a large sample size (*N* > 10,000) and the inclusion of multiple ancestries that are closest to the HRS sample [30–34]. Within each EWAS, regression beta coefficients (β) for significant CpGs reported in the study were used as external weights [21]. Only CpGs that were both reported as significant after Bonferroni correction in the EWAS and present in the HRS DNA methylation data were included in the PEGS. Before PEGS calculation, missing methylation beta values (ranging from 0 to 1) were imputed to the mean methylation beta value of the given CpG for all samples. Each CpG then underwent pre-adjustment for batch effects (plate, plate row, plate column) and white blood cell proportions were estimated using Houseman’s method (NK, B cell, CD4, CD8, MO) [35].

Some of the selected EWASs evaluated DNA methylation as the dependent variable [30,32,33], while others modeled it as the independent variable [31,34]. In this study, we aimed to evaluate the association between the PEGS (independent variable) and the corresponding cardiometabolic risk factor (dependent variable). To ensure the external weight used for each CpG represents the association between DNA methylation and the cardiometabolic risk factor in the EWAS, we transformed the CpG beta coefficients from EWAS that modeled methylation as the dependent variable. For these CpGs, the β (beta coefficient reported in EWAS) was transformed to $\beta^{*}$ (external weight used in this study) using the following formula:

(1) $$R^{2}=\frac{Z^{2}}{Z^{2}+N-1}\;$$

(2) $$\beta^{*}=\beta \times \frac{1-R^{2}}{\left(N-1\right)\times S{E_{\beta }}^{2}}$$

Where:

Z: the Z-score for the CpG-trait association from the EWAS

β: the beta coefficient for the CpG reported in the EWAS

$R^{2}$: the coefficient of determination, calculated using the Z-score

N: the total sample size of the EWAS

SE: the standard error of β for the CpG reported in the EWAS

For each cardiometabolic risk factor, the PEGS for each individual (i=1toN) was then calculated using k CpGs with external weight (w) as:

(3) $$\mathrm{PE}S_{i}=w_{1}m_{i1}+\ldots +w_{k}m_{\mathrm{ik}}$$

Supplemental Table S1 provides the characteristics of selected EWASs, including sample sizes, ancestries, the total number of Bonferroni-significant CpGs, the total number of CpGs identified from the EWAS that were available in HRS for PEGS calculation, the array types for DNA methylation, and the form of external weight used (original or transformed). Prior to analyses, all PEGS were standardized and outliers exceeding 5 SD from the mean were windsorized.

### Genotyping data

Genotyping was performed on HRS participants, with details described elsewhere [36]. Briefly, DNA extracted from saliva samples collected from 2006 to 2012 were genotyped using the Illumina HumanOmni2.5 8v1 and 4v1 arrays. After quality control, genotype data were available for 18,916 participants, and genetic principal components (PCs) were calculated within the full HRS genetic sample. The top two PCs were used in this study to visualize the relationship between genetic ancestry and self-reported race/ethnicity.

### Covariates

All covariates were collected at the time of the blood draw. Sex (male/female), age, and self-reported race/ethnicity (non-Hispanic White, non-Hispanic Black, Hispanic, all other groups) were used as covariates. Educational attainment was classified based on the highest degree the respondent had obtained (less than high school degree, high school degree or equivalent, college degree and above). For the interaction analysis only, we dichotomized educational attainment into less than high school degree vs. high school degree and above. Participants also indicated whether they were using medications for treatment of hypertension, hyperlipidemia, or diabetes at the time of the study.

Smoking was categorized as never smoker, former smoker, and current smoker. For the interaction analysis only, smoking was dichotomized in two ways: current smoker vs. current non-smoker, and never smoker vs. ever smoker (including former and current smokers) to capture the long-term vs. short-term effects of smoking on the associations. Alcoholic drinks per day were calculated by dividing the total units of alcohol consumption per week by seven. According to the National Institute on Alcohol Abuse and Alcoholism (NIAAA) guidelines, for adults 65 years or younger, males consuming more than 2 units per day or females consuming more than 1 unit per day of alcohol are categorized as heavy drinkers. For adults older than 65 years, both males and females consuming more than 1 unit per day were categorized as heavy drinkers. Using these criteria, alcohol consumption status was categorized into three levels based on drinks per day: never drinker (0 drinks/day), moderate drinker (>0 but not meeting criteria for heavy drinker), and heavy drinker. For the interaction analysis only, alcohol consumption status was dichotomized as non-heavy drinker (never drinker and moderate drinker) vs. heavy drinker, given the adverse effects of heavy alcohol use on health, such as increased mortality [37]. Physically active participants were classified as those who engaged in moderate or vigorous activities more than once a week [38].

### Statistical analysis

Correlations among cardiometabolic risk factors and PEGS, respectively, were evaluated using Pearson correlation. Linear regression was used to investigate the association between each PEGS (independent variable) and the corresponding cardiometabolic risk factor (dependent variable). Model 1 adjusted for age, sex, race/ethnicity, educational attainment, and relevant medication use (for SBP/DBP, blood lipids, and fasting glucose only). Model 2 additionally adjusted for smoking, alcohol consumption, and physical activity. All analyses with LDL-C and TG were also adjusted for fasting status, since fasting status can greatly impact these measures. We also conducted stratified analysis to examine these associations within each racial/ethnic group (Model 2). Sensitivity analysis was conducted by incorporating VBS DNA methylation weights (Model 2). For all analyses, *p* < 0.05 was considered significant. The proportion of variance (R^2^) in the cardiometabolic trait attributable specifically to the PEGS, beyond the contribution of adjusted covariates, was calculated for both Models 1 and 2.

For each significant association between a PEGS and a cardiometabolic risk factor, we explored whether the association was modified by demographic factors (age, sex, educational attainment) and health behaviors (smoking, alcohol consumption, physical activity) by introducing interaction terms between each PEGS and the demographic factor or health behavior into Model 2. Age was centered in the interaction analysis between age and PEGS. Educational attainment was analyzed as a two-level variable (less than high school degree vs. high school or above degree). To study the long-term and current impact of smoking on the epigenome, we examined smoking status in two ways [1]: non-current smoker vs. current smoker, and [2] never smoker vs. ever smoker. Alcohol consumption was examined as a dichotomous variable (non-heavy drinker vs. heavy drinker). False discovery rate (FDR) was used to account for multiple testing across the number of interactions examined for each association between a PEGS and its corresponding risk factor, with FDR-q <0.05 considered significant. For significant interactions, associations between the PEGS and corresponding cardiometabolic risk factor were plotted at each level of the categorical factor. For age, the associations are presented at the 25th percentile (61 years) and the 75th percentile (77 years) of the sample. Finally, to determine the range of values for age, a continuous modifier, where the effect of the PEGS on the corresponding cardiometabolic risk factor is statistically significant at *p* < 0.05, we performed a region of significance test using the Johnson-Neyman procedure [39] for each interaction previously identified between age and a PEGS.

All analyses were conducted in R software (version 4.2.3). The *sjPlots* package was used for interaction plots, and the *interactions* package was used for the region of significance tests.

## Results

The mean age of the participants (*N* = 3,996) was 69.5 years, and 58.7% were female (Table 1). The majority of the sample self-reported as non-Hispanic White (66.5%), with 16.3% non-Hispanic Black, 14.1% as Hispanic, and 3.1% as other racial/ethnic groups. A total of 44.5% never smoked, 44.1% were former smokers, and 11.4% were current smokers. Over half (60.8%) of the participants reported not currently drinking, where 30.6% and 8.5% were categorized as moderate drinkers and heavy drinkers. Over half of the participants had educational attainment equivalent to a high school degree (59.2%), and 24.1% had a college degree or beyond. Approximately 47.2% of the respondents self-identified as physically active. We observed positive correlations among the cardiometabolic risk factors (Supplemental Table S2A), except for some of the correlations that included HDL-C or LDL-C. Similarly, most pairs of the PEGS were positively correlated (Supplemental Table S2B). However, PEGS_LDL-C_ and PEGS_HDL-C_ were negatively correlated with the other PEGSs, except for PEGS_HDL-C_ with PEGS_SBP_ and PEGS_DBP_. Supplemental Figure S1 shows the top two genetic principal components (PCs) color-coded by self-reported race/ethnicity for the subset of this study sample who had available genotype data (*n* = 3,512). The PC plot shows that self-reported race/ethnicity groups generally cluster by genetic ancestry (Supplemental Figure 1). However, as has been observed in other US-based studies, Hispanic participants show a heterogeneous genetic ancestry and slightly overlap with both non-Hispanic Whites and African Americans.

**Table 1.** Descriptive statistics of health and retirement study participants (*N* = 3,996).

	Mean (SD) or N (%)
Age (years)	69.5 (9.6)
Female sex	2344 (58.7%)
Race/ethnicity	
Non-Hispanic White	2657 (66.5%)
Non-Hispanic Black	652 (16.3%)
Hispanic	565 (14.1%)
All other groups	122 (3.1%)
Smoking status	
Never smoker	1779 (44.5%)
Former smoker	1763 (44.1%)
Current smoker	454 (11.4%)
Alcohol consumption status	
Nondrinker	2431 (60.8%)
Moderate drinker	1224 (30.6%)
Heavy drinker	341 (8.5%)
Educational attainment	
Less than high school degree	670 (16.8%)
High school degree or equivalent	2364 (59.2%)
College degree and above	962 (24.1%)
Physically active	1885 (47.2%)
Hypertension medication use	1972 (49.3%)
Lipid-lowering medication use	1919 (48.0%)
Diabetes medication use	867 (21.7%)
***Cardiometabolic risk factors***	
SBP (mmHg) (*n* = 3,428)	128.1 (18.6)
DBP (mmHg) (*n* = 3,428)	76.6 (10.7)
BMI (kg/m^2^) (*n* = 3,985)	30.1 (6.7)
CRP (mg/L)^a^ (*n* = 3,984)	4.9 (9.9)
HDL-C (mg/dL)^a^ (*n* = 3,977)	56.9 (18.8)
LDL-C (mg/dL) (*n* = 3,893)	102.3 (35.4)
TG (mg/dL)^a^ (*n* = 3,976)	147.4 (94.3)
Fasting glucose (mg/dL)^a^ (*n* = 2,628)	111.3 (42.4)

SD, standard deviation; SBP, systolic blood pressure; DBP, diastolic blood pressure; BMI, body mass index; CRP, C-reactive protein; HDL-C, high-density lipoprotein; LDL-C, low-density lipoprotein; TG, triglycerides.

CRP, HDL-C, TG, and fasting glucose were natural log-transformed prior to the analysis.

### Associations between poly-epigenetic scores and cardiometabolic risk factors

All PEGS were positively associated with their corresponding cardiometabolic risk factors (Model 1), and all associations remained after further adjustment for health behaviors (Model 2, Table 2). In Model 1, the R^2^ for cardiometabolic risk factor explained by PEGS ranged from 0.005 to 0.137, and these values remained similar after further adjustments. In Model 2, the strongest association was between PEGS_BMI_ and BMI, with every 1-SD increase in PEGS_BMI_ associated with a 2.41 kg/m^2^ increase in BMI (*p* = 2.75 × 10^−124^), followed by the association between PEGS_TG_ and ln(TG) (*p* = 8.46 × 10^−105^) and PEGS_CRP_ and ln(CRP) (*p* = 3.20 × 10^−78^). For a participant with TG or CRP level at the sample mean (mean TG = 147.4 mg/dL; mean CRP = 4.9 mg/L), a 1-SD increase in PEGS_TG_ and PEGS_CRP_ was associated with a 27.31 mg/dL increase in TG and a 1.85 mg/L increase in CRP, respectively. For blood pressure, a 1-SD increase in PEGS_SBP_ and PEGS_DBP_ was associated with an increase of 1.21 and 0.80 mmHg in SBP and DBP, respectively. A 1-SD increase in PEGS_HDL-C_ was associated with a 4.74 mg/dL increase in HDL for a participant with HDL-C level at the sample mean (56.9 mg/dL), and a 1-SD increase in PEGS_LDL-C_ was associated with a 4.24 mg/dL increase in LDL-C. Finally, for a participant with fasting glucose level at the sample mean (111.3 md/dL), a 1-SD increase in PEGS_Fasting glucose_ was associated with an increase of 3.39 mg/dL in fasting glucose.

**Table 2.** Associations between poly-epigenetic scores (PEGS) and corresponding cardiometabolic risk factors.

		Model 1				Model 2			
	Sample size	β^a^	SE	R^2 c^	P	β^a^	SE	R^2 c^	P
SBP	3,428	1.31	0.32	**0.005**	**4.01 × 10**^**−5**^	1.21	0.33	**0.004**	**2.08 × 10**^**−4**^
DBP	3,428	0.91	0.18	**0.007**	**6.96 × 10**^**−7**^	0.80	0.19	**0.005**	**1.78 × 10**^**−5**^
BMI	3,985	2.48	0.10	**0.137**	**4.24 × 10**^**−129**^	2.41	0.10	**0.132**	**2.75 × 10**^**−124**^
CRP	3,984	0.33	0.02	**0.094**	**2.26 × 10**^**−87**^	0.32	0.02	**0.085**	**3.20 × 10**^**−78**^
ln(HDL-C)	3,977	0.09	0.005	**0.082**	**3.02 × 10**^**−76**^	0.08	0.005	**0.067**	**3.80 × 10**^**−62**^
LDL-C^b^	3,893	4.17	0.54	**0.015**	**9.40 × 10**^**−15**^	4.24	0.54	**0.016**	**6.05 × 10**^**−15**^
ln(TG)^b^	3,976	0.16	0.01	**0.109**	**1.69 × 10**^**−101**^	0.17	0.01	**0.113**	**8.46 × 10**^**−105**^
ln(Fasting glucose)	2,515	0.03	0.004	**0.010**	**4.52 × 10**^**−12**^	0.03	0.004	**0.010**	**6.42 × 10**^**−11**^

SBP, systolic blood pressure; DBP, diastolic blood pressure; BMI, body mass index; CRP, C-reactive protein; HDL-C, high-density lipoprotein; LDL-C, low-density lipoprotein; TG, triglycerides.

Model 1: Cardiometabolic risk factor ~ PEGS + age + sex + race/ethnicity + educational attainment (less than high school degree, high school degree or equivalent, college degree and above) + medication use (for SBP/DBP, lipids, and fasting glucose).

Model 2: Model 1 + smoking + alcohol consumption + physical activity.

^a^The beta coefficient corresponds to the change in the corresponding cardiometabolic risk factor with a 1-standard deviation (SD) increase in PEGS.

^b^Models were additionally adjusted for fasting status.

^c^The R^2^ represents the proportion of variance for the cardiometabolic trait attributable to the PEGS beyond the contribution of adjusted covariates.

*P*-value < 0.05 in bold.

When stratified by racial/ethnic groups, all associations were significant in the non-Hispanic White and Hispanic samples, with similar effect estimates for most associations (Supplemental Table S3). The associations were fully attenuated for SBP and DBP in the non-Hispanic Black sample, although this was likely due to sample size since the effect estimate for DBP was similar to the non-Hispanic White sample. After incorporating sample weights and survey design, the associations between the PEGSs and cardiometabolic risk factors remained significant and were similar in magnitude compared to the unweighted associations (Supplemental Table S4).

### Interactions between poly-epigenetic scores and demographic factors on cardiometabolic risk factors

**Figure 1.**
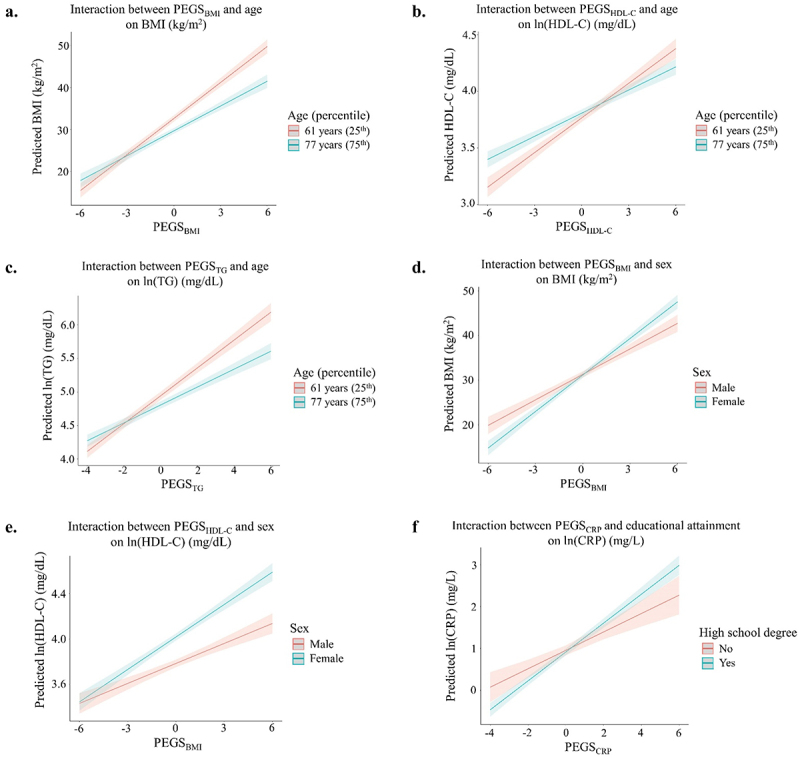
Interaction plots of significant interactions (FDR q < 0.05) between poly-epigenetic scores and demographic factors on cardiometabolic risk factors.

We found significant interactions between age and PEGS_BMI_, PEGS_HDL-C_, and PEGS_TG_ on their corresponding cardiometabolic risk factors, with all associations stronger in younger participants (25th percentile, 61 years) (Table 3 and Figure 1). A 1-SD increase in PEGS_BMI_ was associated with an increase of 2.85 vs. 1.97 kg/m^2^ in BMI in younger vs. older (75th percentile, 77 years) participants (Figure 1a). Because HDL-C and TG were natural log-transformed prior to analysis, the interpretation below applies to an individual who had a lipid level at the sample mean (56.8 mg/dL for HDL-C and 147.4 mg/dL for TG). A 1-SD increase in PEGS_HDL-C_ was associated with an increase of 6.04 vs. 4.06 mg/dL in HDL-C in younger vs. older participants (Figure 1b), and a 1-SD increase in PEGS_TG_ was associated with an increase of 34.68 vs. 20.68 mg/dL in TG in younger vs. older participants (Figure 1c). In addition, the associations between PEGS_BMI_ and PEGS_HDL-C_ on their cardiometabolic risk factors were both stronger in females compared with males. In females, a 1-SD increase in PEGS_BMI_ was associated with an increase of 3.55 kg/m^2^ in BMI, whereas this was only associated with an increase of 2.73 kg/m^2^ in males (Figure 1d). In addition, a 1-SD increase in PEGS_HDL-C_ was associated with an increase of 8.03 vs. 5.67 mg/dL in HDL-C for females vs. males (Figure 1e). Lastly, the association between PEGS_CRP_ and CRP was stronger in those who had a high school degree, as a 1-SD increase in PEGS_CRP_ was associated with an increase of 2.05 vs. 1.21 mg/L in CRP for those with a high school degree vs. those without (Figure 1f).

a: Predicted BMI by PEGS_BMI_ at the 25th (61 years) and 75th percentile (77 years) of age

b: Predicted ln(HDL-C) by PEGS_HDL-C_ at the 25th (61 years) and 75th percentile (77 years) of age

c: Predicted ln(TG) by PEGS_TG_ at the 25th (61 years) and 75th percentile (77 years) of age

d: Predicted BMI by PEGS_BMI_ in male and female

e: Predicted ln(HDL-C) by PEGS_HDL-C_ in male and female

f: Predicted ln(CRP) by PEGS_CRP_ in participants with a high school degree and those without one

PEGS, poly-epigenetic score; BMI, body mass index; HDL-C, high-density lipoprotein; TG, triglycerides; CRP, C-reactive protein

Interaction model: Cardiometabolic risk factor ~ PEGS + age + sex + race/ethnicity + educational attainment + medication use (for hypertension, lipid-lowering, and diabetes) + smoking + alcohol consumption + physical activity + PEGS × demographic factor

Age was scaled for the interaction analysis when examining age as the effect modifier.

Model was additionally adjusted for fasting status for TG analysis.

The line and corresponding confidence intervals represent the predicted level of cardiometabolic risk factor at the corresponding value of PEGS within each level indicated in the legend.

**Table 3.** Significant interactions between poly-epigenetic scores (PEGS) and demographic factors or health behaviors on cardiometabolic risk factors (CMD).

Multiplicative term	CMD risk factor	Sample size	β_PEGS_	P_PEGS_	β_demo/behavior_	P_demo/behavior_	β_Interaction_	P_Interaction_	FDR-q^e^
Demographic factors									
PEGS × Age^a^	BMI	3,985	2.38	**6.59 × 10**^**−123**^	−0.18	**1.01 × 10**^**−67**^	−0.06	**2.12 × 10**^**−8**^	**1.49 × 10**^−^**7**
	(ln)HDL-C	3,977	0.08	**1.07 × 10**^**−66**^	0.003	**7.62 × 10**^**−8**^	−0.002	**1.94 × 10**^**−6**^	**1.36 × 10**^−^**5**
	ln(TG)^f^	3,976	0.17	**1.49 × 10**^**−107**^	−0.01	**5.54 × 10**^**−26**^	−0.005	**3.92 × 10**^**−10**^	**2.74 × 10**^**−9**^
PEGS × Sex^b^	BMI	3,985	1.91	**1.68 × 10**^**−35**^	−0.13	0.503	0.82	**1.69 × 10**^**−5**^	**5.91 × 10**^−^**5**
	(ln)HDL-C	3,977	0.06	**7.35 × 10**^**−17**^	0.23	**5.62 × 10**^**−137**^	0.04	**4.72 × 10**^**−5**^	**1.65 × 10**^−^**4**
PEGS × High school degree^c^	ln(CRP)	3,984	0.22	**3.83 × 10**^**−8**^	−0.04	0.410	0.13	**0.004**	**0.027**
Health behaviors									
*Smoking*									
PEGS × Current smoker^d^	ln(HDL-C)	3,977	0.08	**3.67 × 10**^**−50**^	−0.05	**2.19 × 10**^**−4**^	0.04	**0.004**	**0.009**

PEGS, poly-epigenetic scores; CMD, cardiometabolic risk factors; BMI, body mass index; HDL-C, high-density lipoprotein; TG, triglycerides; CRP, C-reactive protein.

Model: Cardiometabolic risk factor ~ PEGS + age + sex + race/ethnicity + educational attainment + medication use (for hypertension, lipid-lowering, and diabetes) + smoking + alcohol consumption + physical activity + PEGS × hypothesized effect modifier (i.e., demographic factor or health behavior).

PEGS effect sizes (β_PEGS_) correspond to the change in the cardiometabolic risk factor associated with a 1-unit increase in PEGS. Demographic factor/health behavior effect sizes (β_demo/behavior_) correspond to the change in the cardiometabolic risk factor associated with a 1-unit increase in age or with the non-reference level for categorical effect modifiers. Interaction effect sizes (β_Interaction_) correspond to the change in effect of β_PEGS_ on the cardiometabolic risk factor for each 1-unit increase (or level) of the demographic factor/health behavior.

^a^Age was centered in this analysis.

^b^Reference group: male.

^c^Reference group: less than high school degree.

^d^Smoking status was analyzed as a dichotomous variable (current non-smoker vs. current smoker), with current non-smoker as the reference group.

^e^FDR correction was applied to account for multiple testing across the number of interactions examined for each association between a PEGS and its corresponding risk factor.

^f^Model was additionally adjusted for fasting status.

*Only significant interactions (FDR-q < 0.05) were included in the table.

*P*-value < 0.05 in bold.

### Interactions between poly-epigenetic scores and health behaviors on cardiometabolic risk factors

**Figure 2.**
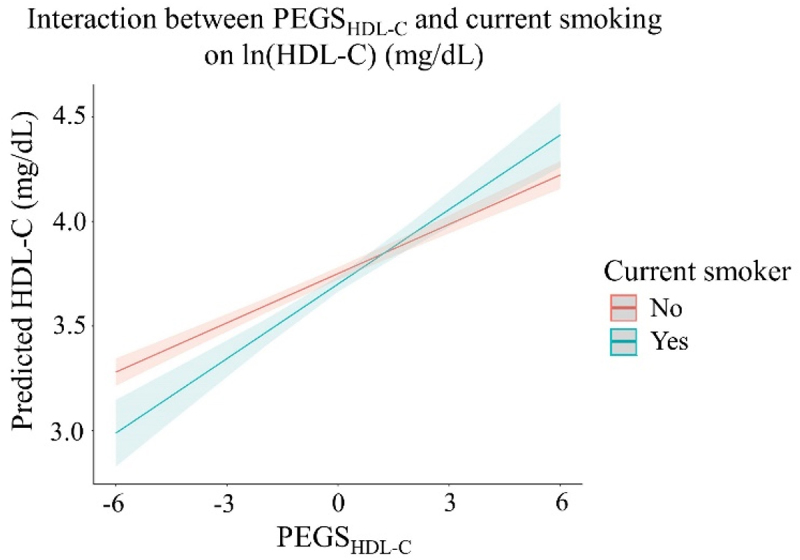
Interaction plots of significant interactions (FDR q < 0.05) between poly-epigenetic scores and health behaviors on cardiometabolic risk factors.

The association between PEGS_HDL-C_ and HDL-C was stronger in those who currently smoke compared to current non-smokers. For someone with HDL-C at the sample mean (56.9 mg/dL), a 1-SD increase in PEGS_HDL-C_ was associated with an increase of 4.43 mg/dL in HDL-C in those who did not currently smoke compared to an increase of 6.87 mg/dL in current smokers (Figure 2). No significant interactions between PEGS and ever smoking, alcohol drinking, or physical activity were detected.

Predicted ln(HDL-C) by PEGS_HDL-C_ in current non-smokers and current smokers

PEGS, poly-epigenetic score; HDL-C, high-density lipoprotein

Interaction model: Cardiometabolic risk factor ~ PEGS + age + sex + race/ethnicity + educational attainment + medication use (for hypertension, lipid-lowering, and diabetes) + smoking + alcohol consumption + physical activity + PEGS × health behavior

Smoking status was categorized as current non-smokers vs. current smokers

The line and corresponding confidence intervals represent the predicted level of cardiometabolic risk factor at the corresponding value of PEGS within each level indicated in the legend.

### Regions of significance tests for age on the associations between poly-epigenetic scores and cardiometabolic risk factors

For all significant interactions that include age (PEGS_BMI_, PEGS_HDL-C_, and PEGS_TG_), the regions of significance tests revealed that the effect of the PEGS on the corresponding cardiometabolic trait was modified by age for all ages at or below 98 years.

## Discussion

In this study, all PEGSs were positively associated with their corresponding cardiometabolic risk factors, with the strongest association observed for BMI. Although significantly associated, the amount of variation in the cardiometabolic risk factors explained by the PEGSs was small to modest (i.e., ranging from 0.4% to 13.2%). Most associations remained consistent across racial/ethnic groups. The associations for BMI and HDL-C were stronger in younger participants and in females. A stronger association for TG was detected in younger participants. Compared to those without a high school degree, participants with a degree had a stronger association between for CRP. Stronger effects were also detected for HDL-C in current smokers compared to current non-smokers. To our knowledge, this study is the first to generate multiple PEGSs by leveraging findings from existing large-scale, racially/ethnically diverse EWASs to validate their associations with corresponding cardiometabolic risk factors in a multi-ethnic/racial cohort of older US adults. We also identified subgroups based on demographic factors or health behaviors where PEGSs might be more powerful.

While there are currently few studies that have examined the associations between PEGSs and cardiometabolic traits, our findings align with existing conclusions. For example, the strongest association that we observed was between PEGS_BMI_ and BMI. Correspondingly, other studies have shown that a large proportion (18%) of variation in BMI variation at population level can be explained by differentially methylated CpGs in blood [40], similar to the variation explained by PEGS_BMI_ in our study (14%). A previous study also showed that a PEGS_BMI_ calculated using regression weights from penalized regression was associated with BMI in an independent cohort, as well as with other cardiometabolic risk factors [23]. However, the generalizability is limited since the samples included only European ancestry participants, and the regression weights were estimated in a relatively small sample (*n* = 2,562). We have also previously shown that similarly constructed PEGS_SBP_ and PEGS_DBP_ are associated with blood pressure in HRS [41]. Our current study differs from the previous one in that we used an updated variable transformation to obtain more accurate effect weights for the PEGSs, accounted for hypertension mediation differently, and tested a larger set of interactions; however, the overall conclusions for blood pressure were similar. Our current study expands on the limited existing literature by constructing PEGSs for multiple cardiometabolic risk factors using weights from large-scale EWAS (all *N* > 10,000) conducted in multiple racial/ethnic groups.

Although all CpGs used for each PEGS were identified from a trait-specific EWAS, not all CpGs are causal or even precursors of the traits of interest. For example, of the 13 CpGs used for PEGS_SBP_ and PEGS_DBP_, bidirectional Mendelian randomization showed that methylation at one CpG affected blood pressure (BP), while BP influences methylation at 3 CpGs [30]. However, although not all the CpGs used in PEGS were causal for the altered levels in cardiometabolic traits, the successful identification of these CpGs from large-scale EWAS still implies their strong potential as surrogate measures for the corresponding traits.

We observed stronger positive associations between PEGSs and their corresponding cardiometabolic traits for BMI, HDL-C, and TG in younger participants. A previous study found that the associations between another form of PEGS, epigenetic clocks, which are designed as a poly-epigenetic measure of biological aging, were also more strongly associated with blood lipid levels in younger participants compared to older participants in HRS [42]. The less pronounced associations in older participants may be explained by the increased pattern of dysregulation of DNA methylation with advanced aging. The number of DNA lesions increases with age [43], as does epigenetic drift [44], which likely both lead to a weakened relationship between methylation patterns and these CVD risk factors at older ages. Nevertheless, future studies are required to elucidate the underlying biological mechanisms.

We detected stronger associations between PEGS_BMI_ and PEGS_HDL-C_ with their corresponding traits in females compared with males. While few studies have formally examined sex as a modifier of associations between PEGSs and cardiometabolic risk factors, there is some evidence of epigenetic relationships with cardiovascular events that differ by sex. For example, DNA methylation at the promoter region of *PLA2G7*, which produces an enzyme associated with coronary heart disease [45], was associated with coronary heart disease in women only, and this association was independent of other well-known CVD risk factors, including age, smoking, hypertension, and diabetes [46]. A separate study found that DNA methylation at the promoter region of *PTX3* was associated with coronary artery disease only in men [47]. These findings suggest that DNA methylation at specific epigenomic regions may have differential effects on cardiovascular risk between sexes. Future studies are needed to better understand the potential pathways underlying these sexually distinct associations.

The positive association between PEGS_HDL-C_ and HDL-C was stronger in current smokers compared to current non-smokers. Smoking is known to greatly affect DNA methylation [48–50], and it is possible that smoking-induced epigenetic changes may lead to lower HDL levels. Finally, the association between PEGS_CRP_ and CRP was stronger in participants with a high school degree compared to those without. This could be partly due to environmental factors or health behaviors having a greater impact on PEGSs among individuals with lower educational attainment. To date, few studies have these relationships using formal interaction analysis, and more research is needed to verify these findings.

This study has notable strengths. First, we selected trait-specific EWASs conducted in multiple racial/ethnic groups with large sample sizes for PEGS calculation to maximize generalizability [51–53]. The weighted analysis further demonstrated strong associations between PEGS and cardiometabolic risk factors in the overall US population. Second, we conducted formal interaction analyses to identify subgroups where the PEGS might be more useful in a precision health context. These findings could provide information for early detection and intervention based on a person’s demographics or health behaviors. Third, the strong associations we detected suggest the potential utility of surrogate epigenetic-based measures in clinical settings. Compared to clinical measurements typically available at the time of a medical appointment, epigenetic information derived from blood samples may better reflect how external factors (e.g., stress, behavioral, and social factors) have shaped an individual’s susceptibility to specific cardiometabolic diseases by altering gene expression levels [54,55]. In contrast, clinical measurements at one time point may not fully reflect these influences due to latent periods. Additionally, clinical measurements tend to have a high degree of variability [56]; for example, BMI may change rapidly and there may be fluctuations in cholesterol levels due to fasting status or mediation use. However, epigenetic surrogates are relatively more stable over time and thus may be more robust reflections of past exposures that continue to affect health [57,58]. As whole-genome sequencing technologies become more accessible, health professionals could consider using PEGS as a proactive tool for early intervention (e.g., through more targeted encouragement of health behavior modifications or pharmaceutical treatments) to slow the progression of cardiometabolic diseases.

However, several limitations should be acknowledged. First, although we used multi-racial/ethnic EWASs for PEGS calculation, the majority of those EWAS participants were of European ancestry, which may limit PEGS performance. Nevertheless, we expect this impact to be minimal, as our race-stratified analysis identified similar effect estimates across racial/ethnic groups. Second, because the DNA methylation and cardiometabolic risk factors were measured concurrently, it was not possible to establish the temporal relationships between PEGSs and their corresponding cardiometabolic risk factors. Third, although we adjusted for smoking status and alcohol consumption status in the analysis, the strong effects of these health behaviors on DNA methylation might not be fully eliminated, potentially biasing our results. Fourth, the variation in cardiometabolic risk factors explained by the PEGS varied by trait and ranged from very small to more modest (range in R^2^ values = 0.4% to 13.2%, Model 2). For at least some of the cardiometabolic traits, further studies are warranted to explore whether these PEGSs are predictive and potentially useful in clinical settings alongside other biomarkers; however, PEGS that explain a very low amount of variability beyond commonly measured demographics and biomarkers are not likely to be clinically useful. Fifth, the association between PEGS and cardiometabolic risk factors may not be fully captured by the linear regression framework, and future studies should explore the potential non-linear relationships of CpGs and cardiometabolic risk factors using advanced analytical methods. Sixth, the results may be impacted by population structure. In this study, we aimed to examine the association between PEGS and cardiometabolic diseases, both of which are likely influenced by genetics as well as social exposures. Although often correlated, genetic ancestry and self-reported race/ethnicity are different concepts. Ancestry reflects genomic similarity, whereas self-reported race/ethnicity is a social construct. While we adjusted for self-reported race/ethnicity, we were not able to adjust for ancestry due to missing genetic data for some participants. Future studies are encouraged to examine these associations by ancestry to more comprehensively examine how the associations between PEGS and cardiometabolic traits may be influenced by population structure. Lastly, the PEGS in this study were calculated using data from large-scale EWASs based on Illumina beadchip arrays. While the Infinium MethylationEPIC BeadChip used in HRS includes pre-selected CpGs enriched in regulatory regions such as enhancers and transcription factor binding sites, over 40% of FANTOM5 enhancers and less than 30% of ENCODE regulatory elements are represented [59]. Furthermore, methylation at other sites, such as CHH and CHG sites, cannot be assessed using beadchip arrays. As whole-genome bisulfite sequencing (WGBS) becomes more accessible, future EWASs will be essential to validate the methylation sites used for PEGS calculation and to further refine the scores.

## Conclusions

In summary, we found strong and positive associations between all PEGSs and their corresponding cardiometabolic risk factors in US older adults, with most associations stable across racial/ethnic groups. For some cardiometabolic traits, the associations were stronger in younger participants, females, participants with higher educational attainment, and those who currently smoked. The results contribute to the growing body of literature on utilizing whole-genome DNA methylation data in the context of precision health. Epigenetic information may better reflect how the external environment shapes an individual’s susceptibility to specific types of cardiometabolic diseases, which may not be accurately reflected by the clinical biomarkers due to potential delays or latent periods. Additionally, the findings from the interaction analyses provide valuable insights for customized early prevention and intervention based on an individual’s demographics or health behaviors. Overall, as an exploratory study, our findings are encouraging as they successfully expand on previous conclusions and provide insights for future research, such as examining the relationships between these PEGSs with other health outcomes beyond the corresponding ones. Replication and extension of our results is required to better inform strategies for promoting precision health practices.

## Supplementary Material

-)Supple tab fig.docx

## Data Availability

Health and Retirement Study (HRS) survey data are publicly available at https://hrs.isr.umich.edu/data-products and can be accessed upon request by registered users who meet security requirements and agree to the specified data use conditions. HRS DNA methylation data are available at the National Institute on Aging Genetics of Alzheimer’s Disease Data Storage Site (NIAGADS, accession number: NG00153).
