# Supplementary material for: Poly-epigenetic scores for cardiometabolic risk factors interact with demographic factors and health behaviors in older US Adults
Source: Epigenetics. 2025 Feb 20;20(1):2469205. doi: 10.1080/15592294.2025.2469205 (PMC11844928; doi:10.1080/15592294.2025.2469205)
Supplement: -)Supple tab fig.docx [file KEPI_A_2469205_SM2506.docx]

**Supplemental Table 1.** Epigenome-wide association studies selected for construction of poly-epigenetic scores (PEGS)

| **CVD risk factor** | **Selected EWAS** | **Sample size** | **Discovery sample (n)** | **Replication sample (n)** | **Number of CpGs selected in EWAS** | **Number of CpGs that were available in HRS** | **Methylation array type** | **Outcome**  **in EWAS** | **Exposure**  **in EWAS** | **Weight form** |
| --- | --- | --- | --- | --- | --- | --- | --- | --- | --- | --- |
| SBP/DBP | Richard et al, 2017  (PMID: 29198723) | 17,010 | EA, AA  (n=9,828) | EA, AA, HA  (n=7,182) | 13 | 13 | 450K | Methylation | SBP/DBP | Transformed |
| BMI | Wahl et al, 2017  (PMID: 28002404) | 10,261 | EA, SAA  (n=5,387) | EA, SAA  (n=4,874) | 187 | 178 | 450K | BMI | Methylation | Original |
| CRP | Wielscher et al, 2022  (PMID: 35504910) | 22,774 | EA AA, SAA (n=22,774) | - | 1,765 | 1,653 | 450K or EPIC | ln(CRP) | Methylation | Original |
| HDL-C | Jhun et al, 2021  (PMID: 34183656) | 16,265 | EA, AA, HA (n=16,265) | - | 110 | 104 | 450K or BeadXpress | Methylation | ln(HDL-C) | Transformed |
| LDL-C | Jhun et al, 2021  (PMID: 34183656) | 16,265 | EA, AA, HA (n=16,265) | - | 39 | 37 | 450K or BeadXpress | Methylation | LDL-C | Transformed |
| TG | Jhun et al, 2021  (PMID: 34183656) | 16,265 | EA, AA, HA (n=16,265) | - | 216 | 201 | 450K or BeadXpress | Methylation | ln(TG) | Transformed |
| Fasting glucose | Liu et al, 2019  (PMID: 31197173) | 16,558 | EA  (n=4,808) | EA, AA, HA (n=11,750) | 5 | 4 | 450K | Fasting glucose | Methylation | Original |

SBP, systolic blood pressure; DBP, diastolic blood pressure; BMI, boss mass index; CRP, C-reactive protein; TG, triglycerides; EA, European Ancestries; AA, African Ancestries; HA, Hispanic Ancestries; SAA, South Asian Ancestries.

**Supplemental Table 2A.** Pearson correlations among cardiometabolic risk factors in the Health and Retirement Study

|  | SBP | DBP | BMI | CRP | HDL-C | LDL-C | TG | Fasting glucose |
| --- | --- | --- | --- | --- | --- | --- | --- | --- |
| SBP | 1.00 |  |  |  |  |  |  |  |
| DBP | 0.64* | 1.00 |  |  |  |  |  |  |
| BMI | 0.07* | 0.17* | 1.00 |  |  |  |  |  |
| CRP | 0.04* | 0.04* | 0.16* | 1.00 |  |  |  |  |
| HDL-C | -0.08* | -0.02 | -0.26* | -0.09* | 1.00 |  |  |  |
| LDL-C | 0.04* | 0.15* | -0.03* | -0.01 | 0.04* | 1.00 |  |  |
| TG | 0.06* | 0.08* | 0.13* | 0.02 | -0.43* | 0.09* | 1.00 |  |
| Fasting glucose | 0.09* | 0.03 | 0.17* | 0.1* | -0.21* | -0.08* | 0.26* | 1.00 |

SBP, systolic blood pressure; DBP, diastolic blood pressure; BMI, boss mass index; CRP, C-reactive protein; HDL-C, high-density lipoprotein; LDL-C, low-density lipoprotein; TG, triglycerides

* P < 0.05

**Supplemental Table 2B.** Pearson correlations among poly-epigenetic scores (PEGS) in the Health and Retirement Study

| **PEGS** | PEGS_SBP_ | PEGS_DBP_ | PEGS_BMI_ | PEGS_CRP_ | PEGS_HDL-C_ | PEGS_LDL-C_ | PEGS_TG_ | PEGS_Fasting glucose_ |
| --- | --- | --- | --- | --- | --- | --- | --- | --- |
| PEGS_SBP_ | 1.00 |  |  |  |  |  |  |  |
| PEGS_DBP_ | 0.98* | 1.00 |  |  |  |  |  |  |
| PEGS_BMI_ | 0.15* | 0.11* | 1.00 |  |  |  |  |  |
| PEGS_CRP_ | 0.32* | 0.27* | 0.74* | 1.00 |  |  |  |  |
| PEGS_HDL-C_ | 0.22* | 0.25* | -0.64* | -0.38* | 1.00 |  |  |  |
| PEGS_LDL-C_ | -0.37* | -0.35* | -0.12* | -0.50* | -0.10* | 1.00 |  |  |
| PEGS_TG_ | 0.69* | 0.68* | 0.44* | 0.54* | -0.04* | -0.39* | 1.00 |  |
| PEGS_Fasting glucose_ | 0.48* | 0.50* | 0.19* | 0.16* | -0.06* | -0.07* | 0.39* | 1.00 |

SBP, systolic blood pressure; DBP, diastolic blood pressure; BMI, boss mass index; CRP, C-reactive protein; HDL-C, high-density lipoprotein; LDL-C, low-density lipoprotein; TG, triglycerides

* P < 0.05

|  | **Overall (n=3,996)^a^** | | | **Non-Hispanic White^b^ (n=2,657)** | | | **Non-Hispanic Black^b^ (n=652)** | | | **Hispanic^b^ (n=565)** | | |
| --- | --- | --- | --- | --- | --- | --- | --- | --- | --- | --- | --- | --- |
|  | **β^c^** | **SE** | **P** | **β^c^** | **SE** | **P** | **β^c^** | **SE** | **P** | **β^c^** | **SE** | **P** |
| SBP | 1.21 | 0.33 | **2.08×10^-4^** | 1.10 | 0.37 | **0.003** | 0.56 | 0.90 | 0.533 | 2.77 | 1.07 | **0.010** |
| DBP | 0.80 | 0.19 | **1.78×10^-5^** | 0.75 | 0.22 | **5.72×10^-4^** | 0.81 | 0.51 | 0.112 | 1.13 | 0.56 | **0.045** |
| BMI | 2.41 | 0.10 | **2.75×10^-124^** | 2.49 | 0.12 | **7.28×10^-96^** | 1.87 | 0.28 | **5.72×10^-11^** | 2.25 | 0.27 | **2.50×10^-16^** |
| ln(CRP) | 0.32 | 0.02 | **3.20×10^-78^** | 0.33 | 0.02 | **8.29×10^-55^** | 0.29 | 0.04 | **5.81×10^-12^** | 0.30 | 0.05 | **7.25×10^-10^** |
| ln(HDL-C) | 0.08 | 0.005 | **3.80×10^-62^** | 0.07 | 0.01 | **1.29×10^-38^** | 0.09 | 0.01 | **1.24×10^-11^** | 0.09 | 0.01 | **1.16×10^-10^** |
| LDL-C^d^ | 4.24 | 0.54 | **6.05×10^-15^** | 3.87 | 0.64 | **1.41×10^-9^** | 4.14 | 1.29 | **0.001** | 4.32 | 1.79 | **0.016** |
| ln(TG)^d^ | 0.17 | 0.01 | **8.46×10^-105^** | 0.17 | 0.01 | **1.32×10^-72^** | 0.14 | 0.02 | **1.14×10^-13^** | 0.20 | 0.02 | **4.11×10^-17^** |
| ln(Fasting glucose)^e^ | 0.03 | 0.004 | **6.42×10^-11^** | 0.02 | 0.005 | **1.82×10^-6^** | 0.03 | 0.01 | **0.021** | 0.06 | 0.02 | **3.60×10^-4^** |

**Supplemental Table 3**. Associations between poly-epigenetic scores (PEGS) and corresponding cardiometabolic risk factors in the full sample and stratified by race/ethnic groups

SBP, systolic blood pressure; DBP, diastolic blood pressure; BMI, body mass index; CRP, C-reactive protein; HDL-C, high-density lipoprotein; LDL-C, low-density lipoprotein; TG, triglycerides

^a^Model: Cardiometabolic risk factor ~ PEGS + age + sex + race/ethnicity + educational attainment (less than high school degree, high school degree or equivalent, college degree and above) + medication use (for SBP/DBP, lipids, and fasting glucose) + smoking + alcohol consumption + physical activity

^b^Model: Cardiometabolic risk factor ~ PEGS + age + sex + educational attainment (less than high school degree, high school degree or equivalent, college degree and above) + medication use (for SBP/DBP, lipids, and fasting glucose) + smoking + alcohol consumption + physical activity

^c^The beta coefficient corresponds to the change in the corresponding cardiometabolic risk factor with 1-standard deviation increase in PEGS.

^d^Models were additionally adjusted for fasting status.

^e^Sample sizes for overall, non-Hispanic White, non-Hispanic Black, and Hispanic: 2515, 1760, 409, and 385.

P-value < 0.05 in bold

**Supplemental** **Table 4**. Associations between poly-epigenetic scores (PEGS) and corresponding cardiometabolic risk factors using HRS sample weights

|  |  | **Unweighted** | | | **Weighted** | | | |
| --- | --- | --- | --- | --- | --- | --- | --- | --- |
|  | **Sample size** | **β^b^** | **SE** | **P** | **Sample size** | **β^b^** | **SE** | **P** |
| SBP | 3,428 | 1.21 | 0.33 | **2.07×10^-4^** | 3,311 | 1.47 | 0.51 | **0.005** |
| DBP | 3,428 | 0.80 | 0.19 | **1.78×10^-5^** | 3,311 | 1.06 | 0.28 | **5.10×10^-4^** |
| BMI | 3,985 | 2.41 | 0.10 | **1.00×10^-122^** | 3,844 | 2.53 | 0.13 | **2.59×10^-23^** |
| ln(CRP) | 3,984 | 0.32 | 0.02 | **3.20×10^-78^** | 3,843 | 0.33 | 0.02 | **1.83×10^-19^** |
| ln(HDL-C) | 3,977 | 0.08 | 0.01 | **3.80×10^-62^** | 3,836 | 0.08 | 0.01 | **5.60×10^-17^** |
| LDL-C^c^ | 3,893 | 4.25 | 0.54 | **5.90×10^-15^** | 3,757 | 4.04 | 0.73 | **2.21×10^-6^** |
| ln(TG)^c^ | 3,976 | 0.17 | 0.01 | **8.46×10^-105^** | 3,835 | 0.19 | 0.01 | **3.28×10^-20^** |
| ln(Fasting glucose) | 2,515 | 0.03 | 0.004 | **6.42×10^-11^** | 2,431 | 0.03 | 0.01 | **3.79×10^-4^** |

SBP, systolic blood pressure; DBP, diastolic blood pressure; BMI, body mass index; CRP, C-reactive protein; HDL-C, high-density lipoprotein; LDL-C, low-density lipoprotein; TG, triglycerides

Model: Cardiometabolic risk factor ~ PEGS + age + sex + race/ethnicity + educational attainment (less than high school degree, high school degree or equivalent, college degree and above) + medication use (for SBP/DBP, lipids, and fasting glucose) + smoking + alcohol consumption + physical activity

^a^DNA methylation sample weight was included in the analyses

^b^The beta coefficient corresponds to the change in the corresponding cardiometabolic risk factor with 1-standard deviation increase in PEGS.

^c^Models were additionally adjusted for fasting status.

P-value < 0.05 in bold


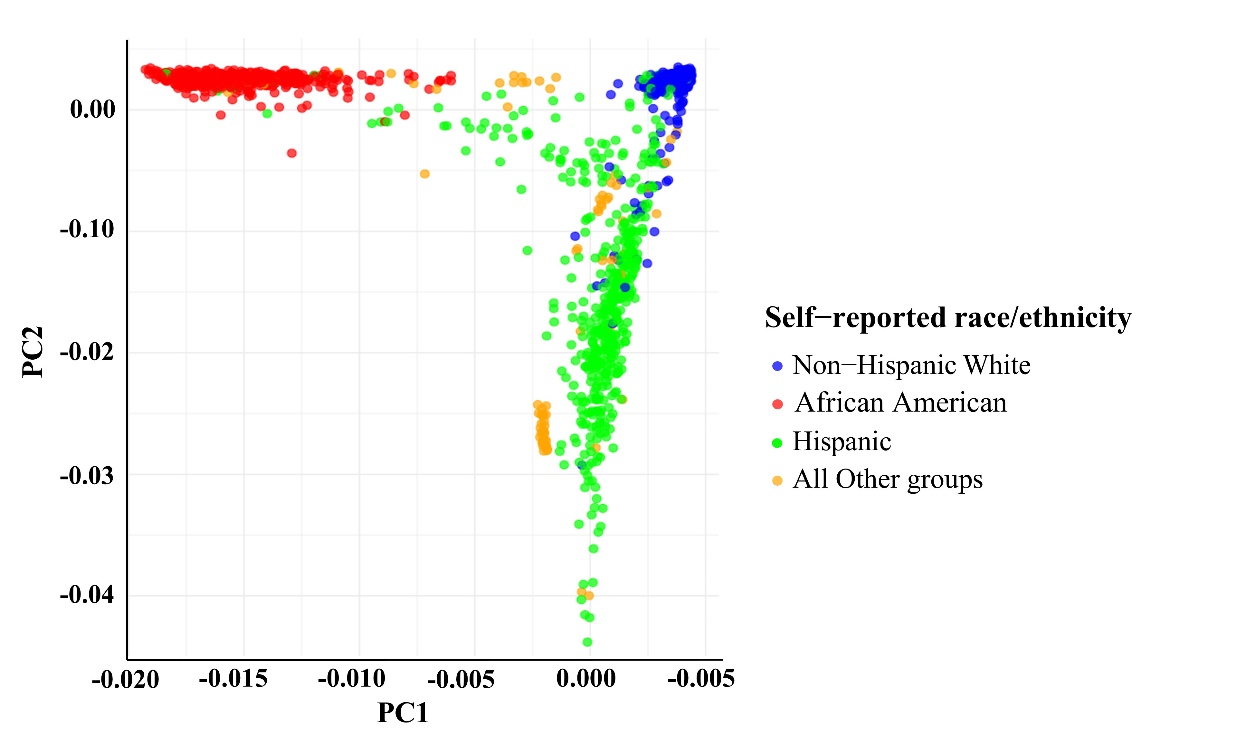


**Supplemental Figure 1.** Scatterplot of the top two genetic principal components (PCs) of study participants color-coded by self-reported race/ethnicity
